# Efficacy and safety of *Limosilactobacillus reuteri* DSM 17938 for the treatment of acute gastroenteritis in children: a systematic review and meta-analysis of randomized controlled trials

**DOI:** 10.3389/fped.2026.1941872

**Published:** 2026-10-16

**Authors:** Ener Cagri Dinleyici, Flavia Indrio, Pedro Gutierrez-Castrellon, Dimas Rosa

**Affiliations:** 1 Department of Pediatrics, Eskisehir Osmangazi University Faculty of Medicine, Eskisehir, Türkiye; 2 Department of Pediatrics, University of Salento, Lecce, Italy; 3 International Scientific Council for Probiotics, Mexico City, Mexico; 4 Grupo de Investigación del Caribe y Centroamérica para la Microbiota, Probióticos y Prebióticos (GICCAMPP), Santa Marta, Colombia

**Keywords:** acute diarrhea, acute gastroenteritis, children, *Lactobacillus reuteri* DSM 17938, *Limosilactobacillus reuteri*, probiotics

## Abstract

**Introduction:**

Acute gastroenteritis (AGE) remains a major global clinical concern in pediatric populations. This systematic review and meta-analysis aimed to evaluate the efficacy and safety of *Limosilactobacillus reuteri* DSM 17938 for the treatment of acute gastroenteritis in children, with the duration of diarrhea as the primary outcome.

**Methods:**

A comprehensive literature search was conducted across MEDLINE (via PubMed), Embase, Scopus, and the Science Citation Index Expanded from inception to April 1, 2026. Two independent reviewers performed study selection, data extraction, and risk of bias assessment using the Cochrane RoB 2 tool. Random-effects models pooled mean differences (MD) and risk ratios (RR), and the certainty of the evidence was appraised via the GRADE approach.

**Results:**

Six randomized controlled trials involving 565 randomized children (527 analyzed for the primary outcome) met the inclusion criteria. *Limosilactobacillus reuteri* DSM 17938 significantly reduced the duration of diarrhea by a mean difference of 0.68 days (95% CI: -1.09 to -0.27). Furthermore, the persistence of diarrhea was significantly lower in the probiotic group at 48 hours (RR 0.45, 95% CI 0.29 to 0.69) and 72 hours (RR 0.41, 95% CI 0.23 to 0.72). No serious or treatment-related adverse events were reported across the trials. The certainty of the evidence was rated as very low for diarrhea duration and low for persistence at 48-72 hours due to high heterogeneity and risk of bias concerns.

**Discussion:**

*Limosilactobacillus reuteri* DSM 17938 produces a modest reduction in the duration of diarrhea and a substantial decrease in persistence at 48-72 hours, accompanied by a favorable safety profile. However, due to the very low certainty of the evidence regarding the primary outcome, these findings should be interpreted with caution in clinical practice.

**Systematic Review Registration:**

https://www.crd.york.ac.uk/PROSPERO/view/CRD420261449562, PROSPERO CRD420261449562.

## Introduction

Acute gastroenteritis (AGE) remains among the most frequent causes of pediatric consultation and hospitalization worldwide, particularly in children under five years of age and in low- and middle-income countries (1). The burden is disproportionately borne by children in low- and middle-income countries, where limited access to clean water, inadequate sanitation, and lower vaccination coverage amplify both incidence and severity (1). Despite important advances in vaccination strategies, oral rehydration therapy (ORT), zinc supplementation, and improved supportive care, diarrheal diseases continue to impose a substantial clinical, economic, and societal burden through increased healthcare utilization, caregiver burden, and impaired child growth and development (1, 2). The COVID-19 pandemic markedly disrupted the epidemiology of pediatric AGE. Although the circulation of major enteric pathogens declined during periods of non-pharmaceutical interventions, several countries subsequently reported a resurgence of rotavirus and other enteric infections following the relaxation of these measures, before gradually returning toward pre-pandemic patterns. These shifts were heterogeneous across regions and were further modified by rotavirus vaccination coverage and by disruptions to surveillance and routine care (3–5).

Beyond the acute diarrheal episode, the intestinal microbiota plays a central role in gut homeostasis, immune modulation, and resistance to enteric pathogens (6, 7). Acute diarrheal events are associated with transient but sometimes profound disruptions of microbial composition, which may prolong symptoms, impair intestinal barrier function, and predispose to recurrence (7). Although public health priorities have historically focused—appropriately—on mortality reduction and cost-effectiveness in the acute phase, these microbiota-related aspects have received comparatively less attention. Growing evidence nonetheless suggests that modulation of the intestinal ecosystem may represent a relevant therapeutic target, particularly in relation to recurrence and nutritional recovery (6).

Probiotics are live microorganisms that, when administered in adequate amounts, confer a health benefit (8). Probiotics have been extensively studied for acute infectious diarrhea and potential clinical benefits of probiotics in pediatric acute infectious diarrhea are a reduction of diarrhea duration, especially effective when started early in the illness. In children who are hospitalized, effective probiotics have been associated with reduced hospital length of stay, approximately 1 day. Most probiotics used for acute infectious diarrhea in studies are well-tolerated with very few adverse effects (9, 10). While probiotics have been extensively studied as adjuncts to rehydration in AGE, their effects are strain- and indication-specific, so evidence for one product cannot be extrapolated to another; each strain must be evaluated individually (9).

Among the most extensively studied strains is *Limosilactobacillus reuteri* (formerly *Lactobacillus reuteri*; derived from the parent strain ATCC 55730 by removal of two antibiotic-resistance plasmids) DSM 17938, a well-characterized human-derived strain (11). *L. reuteri* DSM 17938 has demonstrated immunomodulatory activity, antimicrobial effects (including the production of reuterin), and supportive actions on epithelial barrier function in both experimental and clinical settings (11, 12). *L. reuteri* DSM 17938 is among the most extensively studied probiotic strains in pediatric gastrointestinal disorders and is one of the strains recommended, with varying strength, in international and regional guidelines for AGE (9, 13–18). The World Gastroenterology Organization (WGO) lists *L. reuteri* DSM 17938 among probiotics that may reduce the duration of diarrhea in acute gastroenteritis (17). The ESPGHAN Special Interest Group on Gut Microbiota and Modifications suggests that *L. reuteri* DSM 17938 may be considered, in addition to rehydration therapy, in children with AGE, as it may reduce the duration of diarrhea; however, this remains a conditional recommendation based on very-low-certainty evidence (9). Most recently, the 2025 Ibero–Latin American clinical practice guideline on biotics in pediatric gastroenterology issued an experience-based (Delphi consensus) recommendation for *L. reuteri* in acute diarrhea (18). Notably, the certainty of evidence attached to this strain differs across guidelines, reflecting the limited and heterogeneous underlying trial data.

The most recent quantitative synthesis specific to this strain and indication, published in 2019 (19), pooled four randomized controlled trials (RCTs) (20–23) and reported a reduction in the duration of diarrhea of −0.87 days (95% CI −1.43 to −0.31), with high heterogeneity (I^2^ = 72%) and very-low certainty of evidence; the authors explicitly cautioned that the effect was small and of limited clinical relevance. Since that review, additional double-blind RCTs have been published, including two conducted during and after the COVID-19 pandemic, in emergency-department and outpatient settings outside the previously dominant European, mostly inpatient trial base (24, 25).

An updated and more comprehensive synthesis is therefore both timely and warranted — to incorporate the newly published trials, to re-examine the magnitude and robustness of the effect in the context of a changing AGE epidemiology, and to strengthen the previous analysis with a full time-course of diarrhea persistence. The aim of this systematic review and meta-analysis was to assess the efficacy and safety of *L. reuteri* DSM 17938, compared with placebo or no treatment (both with rehydration therapy), for the treatment of acute gastroenteritis in children, with the duration of diarrhea as the primary outcome.

## Methods

### Protocol and registration

This systematic review and meta-analysis were conducted and reported in accordance with the Preferred Reporting Items for Systematic Reviews and Meta-Analyses (PRISMA) 2020 statement (26) and the Cochrane Handbook for Systematic Reviews of Interventions (27). It updates a previous four-trial meta-analysis of *L. reuteri* DSM 17938 for the treatment of acute gastroenteritis (AGE) in children by incorporating trials published since the last search. The protocol was registered in PROSPERO (registration number: CRD420261449562). No ethical approval was required, as the study used previously published aggregate data.

### Studies were selected using the PICOS framework

#### Population

Children (aged ≤18 years) with acute gastroenteritis, defined as an acute onset of diarrhea (typically ≥3 loose or watery stools per 24 h) lasting less than 14 days.

#### Intervention

*Limosilactobacillus reuteri* (formerly *Lactobacillus reuteri*) DSM 17938, as a single ingredient at any dose, administered with rehydration therapy.

#### Comparator

Placebo or no treatment, in addition to rehydration therapy.

#### Outcomes

The primary outcome was the duration of diarrhea. Secondary outcomes were the proportion of children with persisting diarrhea at 24, 48, 72, 96 and 120 h, duration of hospitalization, mean number of watery stools per day, and adverse events.

#### Study design

Randomized controlled trials (RCTs) only.

Trials evaluating the parent strain *L. reuteri* ATCC 55730, other *L. reuteri* strains, multi-strain products, or *L. reuteri* DSM 17938 for the prevention of antibiotic-associated diarrhea were excluded, as were factorial designs and interventions not comparable with the predefined PICO. Reviews, letters, conference abstracts, and non-randomized studies were not eligible.

### Search strategy

We systematically searched MEDLINE (via PubMed), Embase, Scopus, and the Science Citation Index Expanded (Web of Science) from inception to 1 April 2026. The search combined controlled vocabulary and free-text terms for the intervention (*Lactobacillus reuteri*, *Limosilactobacillus reuteri*, DSM 17938), the condition (diarrhea, diarrhoea, gastroenteritis), and the population (child, infant, pediatric), using database-appropriate syntax and field tags (title, abstract, and keywords). No language or date restrictions were applied. The complete search strategy for each database, including the exact search strings, search dates, and numbers of records retrieved, is provided in Supplementary Table S1. In addition, we screened ClinicalTrials.gov for completed or unpublished trials and hand-searched the reference lists of the included studies and of relevant previous systematic reviews. Google Scholar was searched as a supplementary source for grey literature; because it does not support structured Boolean or field-specific searching, the first 200 records sorted by relevance were screened, in line with common practice for its use in systematic reviews.

### Study selection

Records were de-duplicated and screened in two stages by two independent reviewers (ECD and DR). Titles and abstracts were screened first, followed by full-text assessment of potentially eligible reports against the eligibility criteria. Disagreements were resolved by discussion or by consultation with other reviewers (FI and PGC). The study selection process is summarized in a PRISMA 2020 flow diagram (Figure 1).

**Figure 1:**
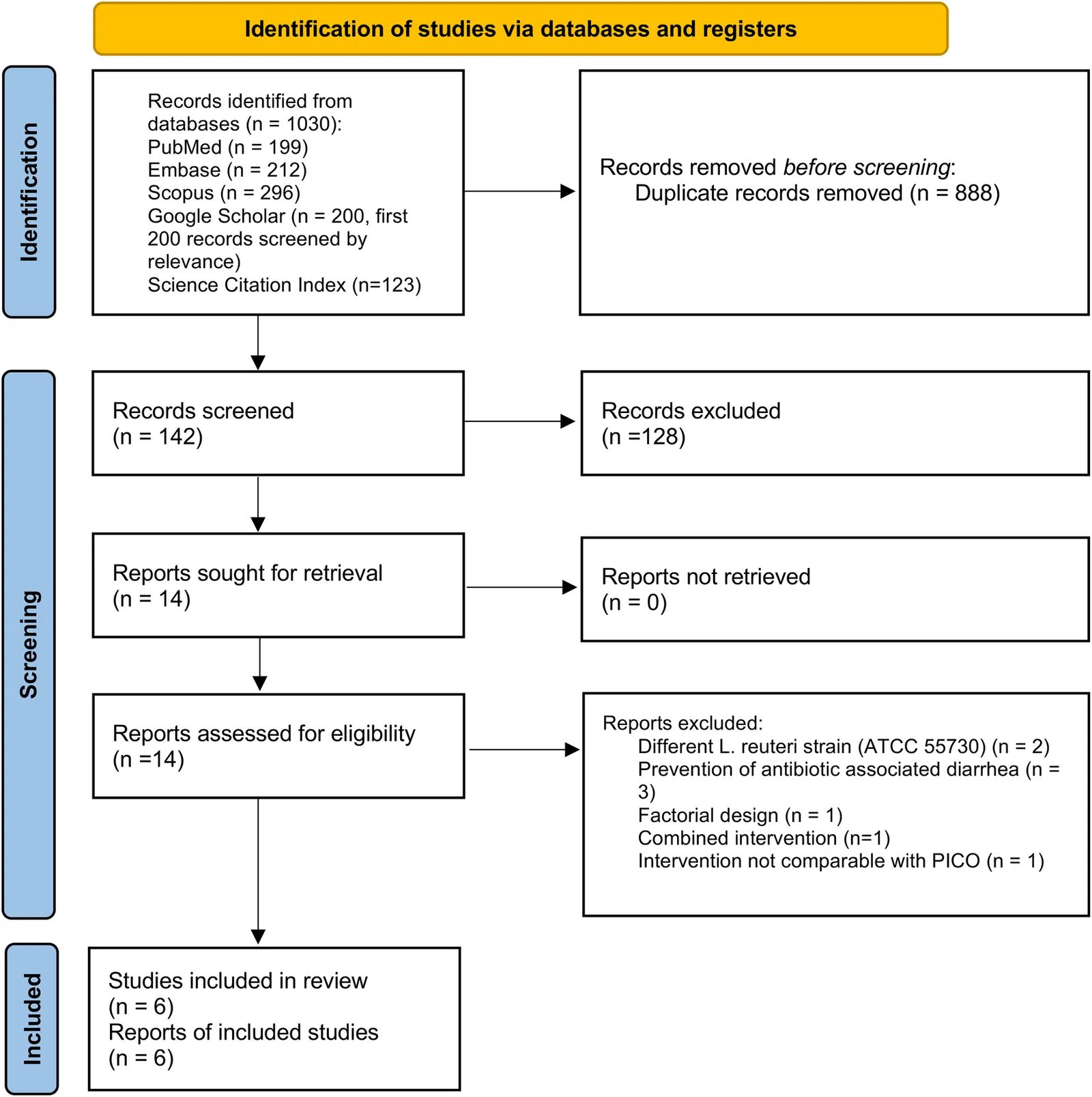
PRISMA 2020 flow diagram of the literature search and study selection process. Records identified through database searching were screened after duplicate removal, followed by full-text eligibility assessment. Reasons for full-text exclusion are presented, and the final number of studies included in the qualitative and quantitative (meta-analysis) synthesis is shown.

### Data extraction

Data were extracted independently by two reviewers (ECD and DR) using a standardized, piloted form. Extracted items included study identifiers (first author, year, country, registration number, enrollment period), design and setting, blinding, participant characteristics (age, sample size analyzed, inclusion and exclusion criteria), intervention details (dose and duration), primary and secondary outcome definitions and time points, funding, and conflicts of interest. For continuous outcomes, we extracted the mean, standard deviation (SD) and number of participants per group; for dichotomous outcomes, the number of events and participants per group. Discrepancies were resolved by consensus. Where duration of diarrhea was reported in hours, values were converted to days. Two trials reported the outcome as median with interquartile range; the mean and SD were estimated from the median, interquartile range and sample size using the method described by Wan et al. (28). Reproduction of the previously published four-trial pooled estimate was used to verify the extraction and analysis pipeline before incorporating the newly identified trials.

### Risk of bias assessment

The risk of bias for the primary outcome was assessed with the Cochrane Risk of Bias 2 (RoB 2) tool, which evaluates five domains: bias arising from the randomization process (D1), deviations from intended interventions (D2), missing outcome data (D3), measurement of the outcome (D4), and selection of the reported result (D5) (29). Domain 2 was assessed under the effect-of-assignment (intention-to-treat) framework. Assessments were performed by two independent reviewers (ECD and DR), with disagreements resolved by consensus. Each trial received a domain-level and an overall judgment of low risk, some concerns, or high risk of bias (Supplementary Figure S1).

### Statistical analysis

Meta-analyses were performed in R version 4.6.1 (2026-06-24) using the *meta* package (version 8.5.0), applying a random-effects model with the DerSimonian–Laird estimator of between-study variance (*τ*^2^). The DerSimonian–Laird estimator was selected as the primary method to maintain methodological consistency with the previous meta-analysis on this strain and indication and with related pediatric probiotic syntheses, allowing direct comparison of pooled estimates across analyses. Because this estimator may underestimate between-study variance when the number of trials is small, we prespecified a sensitivity analysis using the restricted maximum likelihood (REML) estimator with the Hartung–Knapp adjustment, which yields more conservative confidence intervals under these conditions; concordance between the two approaches was used to assess the robustness of the primary estimate. Continuous outcomes were pooled as the mean difference (MD) using inverse-variance weighting; dichotomous outcomes were pooled as the risk ratio (RR) using the Mantel–Haenszel method. For the diarrhea-persistence outcome, a risk ratio below 1 favors *L. reuteri* DSM 17938. Results are reported with 95% confidence intervals (CIs). A two-sided *p* < 0.05 was considered statistically significant. Statistical heterogeneity was quantified using the I^2^ statistic and the *τ*^2^ estimate, with the *χ*^2^ (Q) test; I^2^ values of approximately 25%, 50% and 75% were interpreted as low, moderate and high heterogeneity, respectively. A prediction interval was calculated for the primary outcome. Because fewer than 10 studies were available for each outcome, publication bias was not assessed formally, as funnel-plot–based methods are unreliable in this setting.

### Subgroup and sensitivity analyses

Subgroup analyses were prespecified but, given the small number of trials, were considered exploratory. For the primary outcome, prespecified subgroup analyses were performed according to blinding (single- vs. double-blind), clinical setting (inpatient vs. outpatient/emergency), and probiotic dose. Between-subgroup differences were assessed with the test for subgroup differences. Sensitivity analyses examined (i) the influence of the two median-reported trials by excluding them and by replacing the converted values with author-reported SDs where available, and (ii) the influence of the between-study variance estimator by repeating the primary analysis with the REML estimator and the Hartung–Knapp adjustment; the primary effect was re-estimated accordingly under each scenario.

### Certainty of evidence

The certainty of evidence for each outcome was rated using the Grading of Recommendations, Assessment, Development and Evaluation (GRADE) approach, considering within-study risk of bias, inconsistency, indirectness, imprecision, and publication bias (30). Findings were summarized in a GRADE evidence profile/Summary of Findings table.

### Use of large language models

A large language model (Claude, Anthropic) was used under human supervision to assist with language editing, improving flow, and checking internal consistency. It was not used for study selection, data extraction, risk-of-bias assessment, statistical analysis, or interpretation of the evidence. All AI-assisted outputs were reviewed and verified by the authors, who take full responsibility for the content.

## Results

### Study selection

The database search identified 1030 records. After removal of duplicates (*n* = 888) and screening of *n* = 142 titles and abstracts, *n* = 14 full-text reports were assessed for eligibility. Following full-text assessment, eight reports were excluded (different *L. reuteri* strain, ATCC 55730, *n* = 2; prevention of antibiotic-associated diarrhea, *n* = 3; factorial design, *n* = 1; combined intervention, *n* = 1; intervention not comparable with PICO, *n* = 1) (31–38). Six RCTs (20–25) met the inclusion criteria and were included in both the qualitative and quantitative synthesis (Figure 1, PRISMA flow diagram).

### Characteristics of included studies

The characteristics of the six included trials are summarized in Table 1. The trials were conducted in Italy, Türkiye (two trials), Poland, Paraguay, and Thailand, and randomized a total of 565 (279 in the *L. reuteri* group and 286 in the control group) children aged between 3 months and 5 years, of whom 527 (257 in the *L. reuteri* group and 270 in the control group) were analyzed for the primary outcome. Three trials were conducted In children who are hospitalized and three in outpatient or emergency-department settings. Four trials were double-blind and placebo-controlled, and two were single-blind. The daily dose of *L. reuteri* DSM 17938 ranged from 1 × 10⁸ to 4 × 10⁸ CFU, administered for 5 days in all trials except one (7 days). In all trials, *L. reuteri* DSM 17938 was given in addition to rehydration therapy, and the comparator was placebo (four trials) or no treatment (two trials). Two of the six trials (24, 25) were conducted during and after the COVID-19 pandemic.

**Table 1:** Characteristics and eligibility criteria of the included studies.

Study	Year	Country	Design/blinding	Setting	Enrollment period	N (LR/Ctrl)*	Age range	Dose/duration	Primary endpoint	Registration/Funding/COI
Dinleyici et al. (20)	2014	Türkiye (multicenter)	RCT, multicentre, single-blind	Hospitalized	Not stated (accepted 2014)	64/63	3–60 mo	1 × 10⁸ CFU/day; 5 days	Duration of diarrhea (hours)	NCT01927094; investigator-initiated; no industry funding; authors declared no conflict of interest related to the study product
**Inclusion:** Age 3–60 mo; acute watery diarrhea lasting 12–72 h; mild–moderate dehydration; hospitalized						**Exclusion:** Hypovolemic shock/ICU need; antibiotics or probiotics within 1 mo; malnutrition (weight <P3); chronic/immunocompromising disease				
Dinleyici et al. (21)	2015	Türkiye (multicenter)	RCT, multicenter, single-blind	Outpatient	Not stated (accepted 2014)	29/31	3–60 mo	1 × 10⁸ CFU/day; 5 days	Duration of diarrhea (hours)	NCT01927094; investigator-initiated; no industry funding; authors declared no conflict of interest related to the study product
**Inclusion:** Age 3–60 mo; acute watery diarrhea (≥3 loose/watery stools/day) lasting 12–72 h; outpatient						**Exclusion:** Need for hospitalization; antibiotics or probiotics within 1 mo; severe malnutrition; severe chronic/immunocompromising disease				
Francavilla et al. (22)	2012	Italy (3 hospitals)	RCT, double-blind, placebo-controlled	Hospitalized	Jan–Jul 2009	35/34	6–36 mo	4 × 10⁸ CFU/day; 7 days	Unresolved diarrhea at day 3; duration of diarrhea	Not registered, no funding declared; no conflicts of interest declared
**Inclusion:** Age 6–36 mo; acute diarrhea (≥2 watery stools/24 h, ≤7 days); mild–moderate dehydration; hospitalized						**Exclusion:** Chronic disease; bloody stools; current antibiotic/probiotic/antidiarrheal use; bacterial cause; need for parenteral rehydration				
Szymański et al. (23)	2019	Poland (1 hospital)	RCT, double-blind, placebo-controlled	Hospitalized	Jan 2017–Nov 2018	44/47	<5 y	2 × 10⁸ CFU/day; 5 days	Duration of diarrhea	NCT02989350; no external funding; study product provided by BioGaia (manufacturer had no role in design, conduct, analysis, or interpretation); one author disclosed serving as a speaker for BioGaia
**Inclusion:** Age <5 y; AGE (change in stool consistency and/or ≥3 stools/24 h) lasting ≤5 days						**Exclusion:** Antibiotics, gelatin tannate, diosmectite, probiotics, racecadotril or zinc within 1 wk; exclusive breast-feeding; chronic GI disease; immunodeficiency; malnutrition				
Iramain et al. (24)	2025	Paraguay (1 center)	RCT, double-blind, placebo-controlled	Emergency dept.	Oct 2021–Jan 2023	62/70	1–60 mo	2 × 10⁸ CFU/day; 5 days	Duration of diarrhea (days)	Not stated (IRB 421/2021) no funding reported; authors declared no conflicts of interest
**Inclusion:** Age 1–60 mo; AGE (<14 days) with no or mild–moderate dehydration						**Exclusion:** Chronic GI/malabsorptive disease; celiac; cystic fibrosis; immunodeficiency; severe malnutrition; antibiotics/antidiarrheals/probiotics within 2 wk; severe dehydration/parenteral hydration				
Rerksuppaphol and Rerksuppaphol (25)	2025	Thailand (1 hospital)	RCT, double-blind, placebo-controlled	Outpatient	Oct 2020–Jul 2021	23/25	1–59 mo	1 × 10⁸ CFU/day; 5 days	Duration of diarrhea (time to resolution)	TCTR20201005005; funded by a research grant from the Faculty of Medicine, Srinakharinwirot University (non-industry); no conflicts of interest declared
**Inclusion:** Age 1–59 mo; acute diarrhea (≥3 loose/watery stools/24 h, <7 days); no or mild dehydration; outpatient						**Exclusion:** Severe dehydration; major systemic illness; chronic GI disease; immunodeficiency; recent antibiotics/probiotics; antidiarrheals within 2 wk; severe malnutrition				

LR, *L. reuteri*; Ctrl, control; AGE, acute gastroenteritis; GI, gastrointestinal; mo, months; y, years; wk, week; CFU, colony-forming units; IRB, institutional review board.

*A total of 565 children were randomized (279 in the *L. reuteri* group and 286 in the control group); the numbers shown are those analyzed for the primary outcome (527 in total: 257 and 270, respectively). Inclusion and exclusion criteria were summarized from each trial's methods section.

### Risk of bias

The risk-of-bias assessment for the primary outcome (duration of diarrhea) is presented in Figure 2 (RoB 2 traffic-light plot). No trial was judged to be at high risk of bias. Five trials were rated as having some concerns: the two single-blind trials by Dinleyici et al. (20, 21), in which outcome ascertainment was only partially blinded (blinded nurses during the hospitalized phase but unblinded caregivers during outpatient follow-up), giving some concerns for deviations from intended interventions (Domain 2) and measurement of the outcome (Domain 4); and Francavilla et al. (22), which was double-blind but not prospectively registered (Domain 5). Iramain et al. (24) was rated as having some concerns owing to the absence of an identified prospective registration, Rerksuppaphol and Rerksuppaphol (25) et al. owing to missing-data handling and the outpatient, caregiver-reported outcome. Szymański et al. (23) was at low risk across all domains. Randomization (Domain 1) and missing-outcome-data (Domain 3) were at low risk in all but one trial.

**Figure 2:**
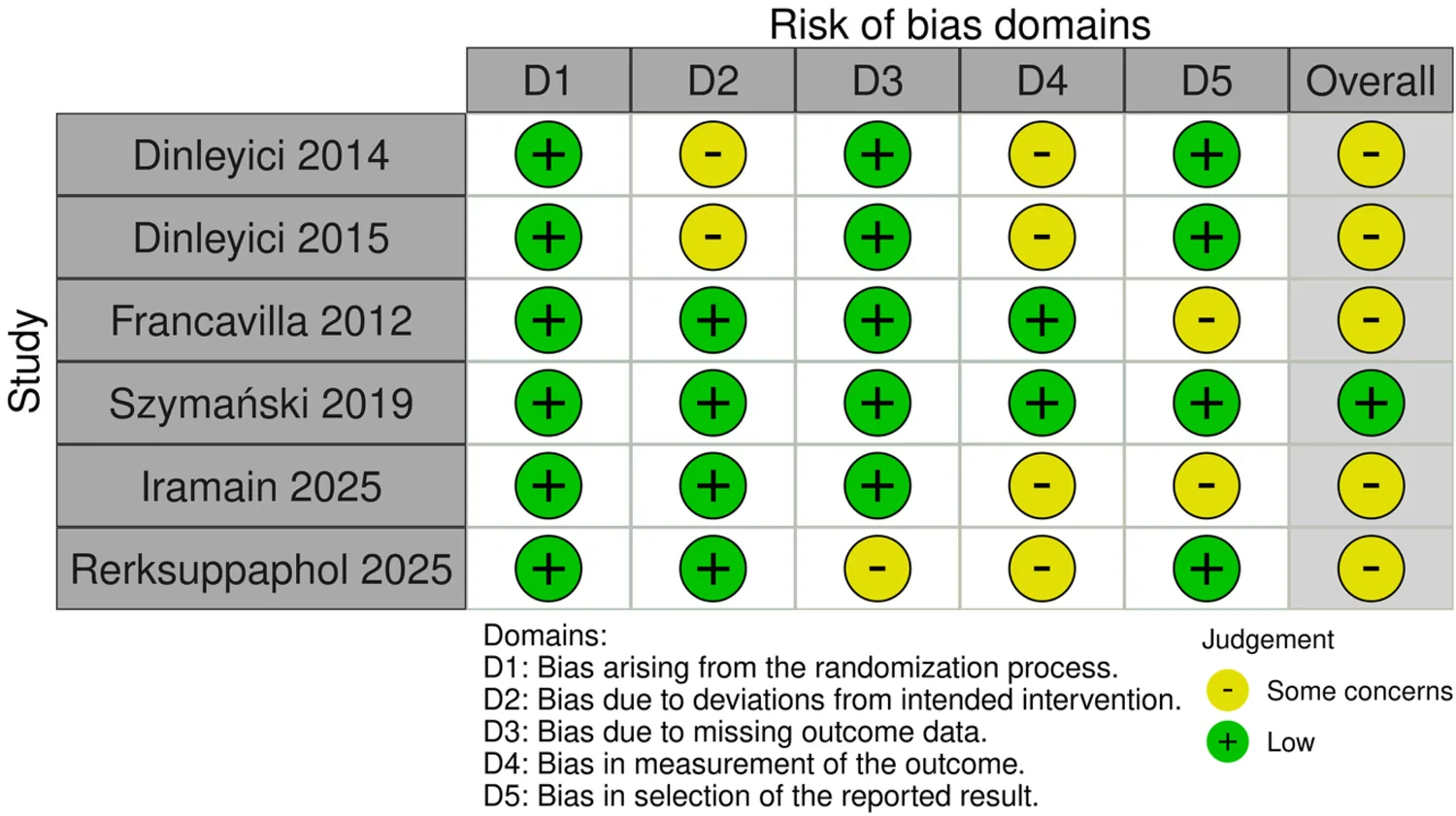
(Rob 2 traffic-light plot). Risk of bias was assessed using the Cochrane Risk of Bias 2 (RoB 2) tool for the primary outcome (duration of diarrhea) and visualized using robvis. Five domains were evaluated: bias arising from the randomization process (D1), deviations from intended interventions (D2), missing outcome data (D3), measurement of the outcome (D4), and selection of the reported result (D5). Most domains were judged as low risk, with some concerns identified across D2, D3, D4, and D5. Overall, five studies were judged to have some concerns, and one study was judged to be at low risk of bias.

### Primary outcome: duration of diarrhea

All six trials reported the duration of diarrhea, and all favored *L. reuteri* DSM 17938. Compared with placebo or no treatment, *L. reuteri* DSM 17938 significantly reduced the duration of diarrhea by a mean difference (MD) of −0.68 days (95% CI −1.09 to −0.27; *p* = 0.001), corresponding to a reduction of approximately 16 h (Figure 3). Statistical heterogeneity was high (I^2^ = 79%, *τ*^2^ = 0.19; Q = 23.9, df = 5, *p* < 0.001). The result was consistent when the analysis was repeated using the REML estimator with the Hartung–Knapp adjustment (MD −0.68 days, 95% CI −1.18 to −0.18). A cumulative meta-analysis showed that the pooled estimate moved gradually toward the null as larger, better-blinded trials accrued, from approximately −1.35 days after the two earliest trials to −0.68 days after inclusion of the two 2025 trials.

**Figure 3:**
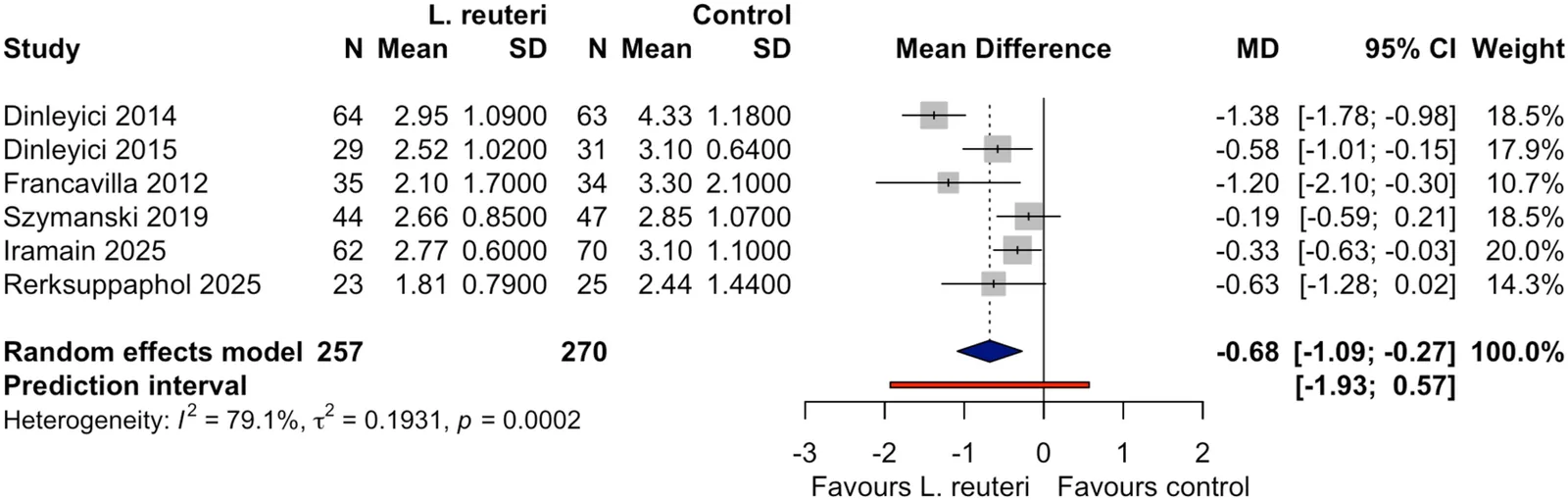
Forest plot of the effect of *Limosilactobacillus reuteri* DSM 17938 on the duration of diarrhea in children with acute gastroenteritis. Forest plot showing the pooled mean difference (MD) in diarrhea duration between children receiving L. reuteri DSM 17938 and those receiving placebo or standard treatment. Negative mean differences favor L. reuteri DSM 17938, indicating a shorter duration of diarrhea. Squares represent the effect estimate for each randomized controlled trial, with the size of each square proportional to the study weight. Horizontal lines indicate 95% confidence intervals (CIs). The diamond represents the pooled effect estimate obtained using a random-effects model, with its width corresponding to the 95% CI. The red horizontal bar indicates the 95% prediction interval. Overall, L. reuteri DSM 17938 significantly reduced the duration of diarrhea compared with the control group (MD, −0.68 days; 95% CI, −1.09 to −0.27), although substantial between-study heterogeneity was observed (I² = 79.1%).

### Sensitivity analyses

The pooled estimate was robust across all sensitivity analyses. Replacing the Wan-estimated standard deviation with the author-reported standard deviation for the Szymański et al. trial resulted in a mean difference (MD) of −0.72 days (95% CI, −1.14 to −0.29). Excluding both trials that reported medians yielded an MD of −0.83 days (95% CI, −1.39 to −0.28). Restricting the analysis to the original four trials included in the previous meta-analysis reproduced the published estimate (MD −0.87 days, 95% CI, −1.43 to −0.31; I^2^ = 72%), confirming the consistency of the data extraction and analytical approach. Analyses using the REML estimator with the Hartung–Knapp adjustment produced nearly identical effect estimates (primary analysis: MD −0.68 days, 95% CI, −1.18 to −0.18; author-reported SD: MD −0.72 days, 95% CI, −1.22 to −0.22; excluding both median-reported trials: MD −0.83 days, 95% CI, −1.64 to −0.02; original four trials: MD −0.87 days, 95% CI, −1.74 to −0.01). The close concordance between the DerSimonian–Laird and the more conservative Hartung–Knapp results indicate that the primary estimate was not an artifact of underestimated between-study variance despite the small number of trials. Across all analyses, the reduction in diarrhea duration remained statistically significant, with the pooled effect consistently below one day. Across all analyses, the reduction in diarrhea duration remained statistically significant, with the pooled effect consistently below one day.

### Subgroup analyses

These prespecified subgroup analyses should be interpreted as exploratory, given the small number of trials and the limited power of the test for subgroup differences. Prespecified subgroup analyses suggested that the treatment effect varied according to study characteristics, although no statistically significant subgroup differences were detected. The pooled effect was larger in the two single-blind trials (MD −0.99 days, 95% CI −1.77 to −0.20; I^2^ = 86%) than in the four double-blind trials (MD −0.42 days, 95% CI −0.72 to −0.12; I^2^ = 37%), with the latter demonstrating both a smaller treatment effect and substantially lower heterogeneity (test for subgroup differences, *p* = 0.19). A similar pattern was observed according to study setting, with a greater reduction in diarrhea duration among children requiring hospitalization (MD −0.90 days, 95% CI −1.78 to −0.02; I^2^ = 89%) than among outpatients or children treated in emergency departments (MD −0.44 days, 95% CI −0.67 to −0.21; I^2^ = 0%). No consistent dose–response relationship was identified. Although the largest pooled effect was observed with the lowest evaluated dose (1 × 10⁸ CFU/day; MD −0.89 days, 95% CI −1.45 to −0.33), this finding should be interpreted cautiously because dose was closely confounded with study identity and blinding. The two-trial subgroup receiving 2 × 10⁸ CFU/day showed a smaller but statistically significant effect (MD −0.28 days, 95% CI −0.52 to −0.04; I^2^ = 0%), whereas the single trial using 4 × 10⁸ CFU/day reported an MD of −1.20 days (95% CI −2.10 to −0.30).

### Secondary outcomes

#### Diarrhea persistence over time

The proportion of children with persisting diarrhea [risk ratio (RR) < 1 favoring *L. reuteri* DSM 17938] was evaluated at successive time points. Szymański et al. (23) did not report this outcome in a usable dichotomous format and was therefore excluded from these analyses [k = 5 at 24–72 h; k = 4 at 96 h and k = 3 at 120 h, as Rerksuppaphol et al. (25) reported outcomes only up to 72 h and one additional trial contributed no events in either arm at 120 h or was therefore not estimable]. A modest reduction in diarrhea persistence was observed at 24 h (RR 0.87, 95% CI 0.80–0.95; I^2^ = 34%). The treatment effect became substantially greater at 48 h (RR 0.45, 95% CI 0.29–0.69; I^2^ = 74%) and reached its maximum at 72 h (RR 0.41, 95% CI 0.23–0.72; I^2^ = 75%). Although fewer studies contributed data beyond 72 h, the beneficial effect remained statistically significant at both 96 h (RR 0.47, 95% CI 0.23–0.95; I^2^ = 71%) and 120 h (RR 0.47, 95% CI 0.30–0.73; I^2^ = 18%). These time-specific estimates were derived from separate meta-analyses at each time point rather than from a single longitudinal model, and the contributing trials were not identical across time points (k = 5 at 24–72 h and k = 4 at 96–120 h). With this caveat, the cross-sectional pattern is consistent with a treatment effect that is largest during the first 48–72 h after treatment initiation and attenuates thereafter, rather than a directly measured change in effect over time (Figures 4–6).

**Figure 4:**
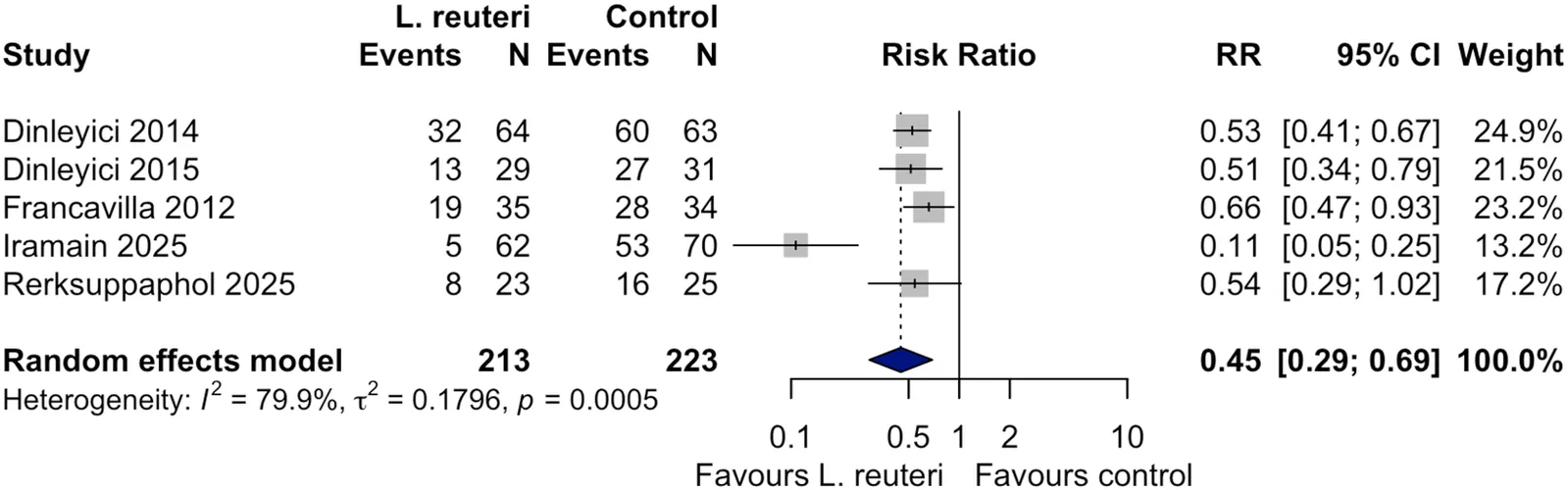
Forest plot of the effect of *Limosilactobacillus reuteri* DSM 17938 on diarrhea persistence at 48 h. The pooled analysis demonstrated a significantly lower risk of persistent diarrhea in the L. reuteri group compared with the control group (RR 0.45, 95% CI 0.29–0.69; random-effects model). Squares represent study-specific risk ratios, with sizes proportional to study weights; horizontal lines indicate 95% confidence intervals, and the diamond represents the pooled effect estimate. Values <1 favor L. reuteri DSM 17938.

**Figure 5:**
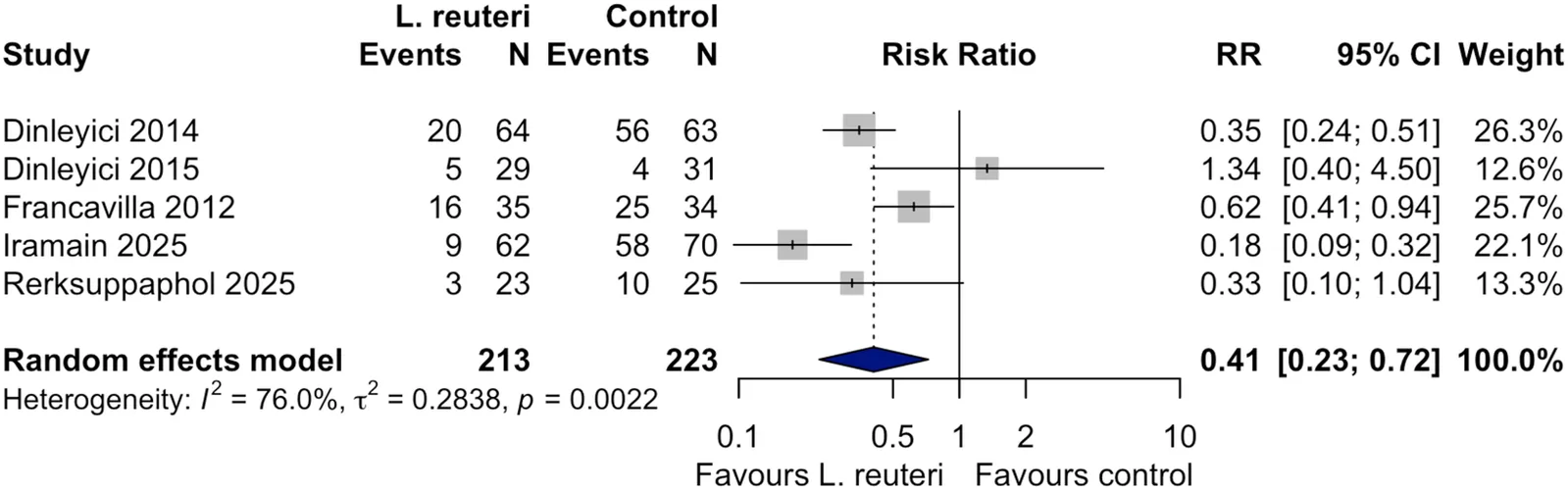
Forest plot of the effect of *Limosilactobacillus reuteri* DSM 17938 on diarrhea persistence at 72 h. The pooled analysis demonstrated a significantly lower risk of persistent diarrhea in the L. reuteri group compared with the control group (RR 0.41, 95% CI 0.23–0.72; random-effects model). Squares represent study-specific risk ratios, with sizes proportional to study weights; horizontal lines indicate 95% confidence intervals, and the diamond represents the pooled effect estimate. Values <1 favor L. reuteri DSM 17938.

**Figure 6:**
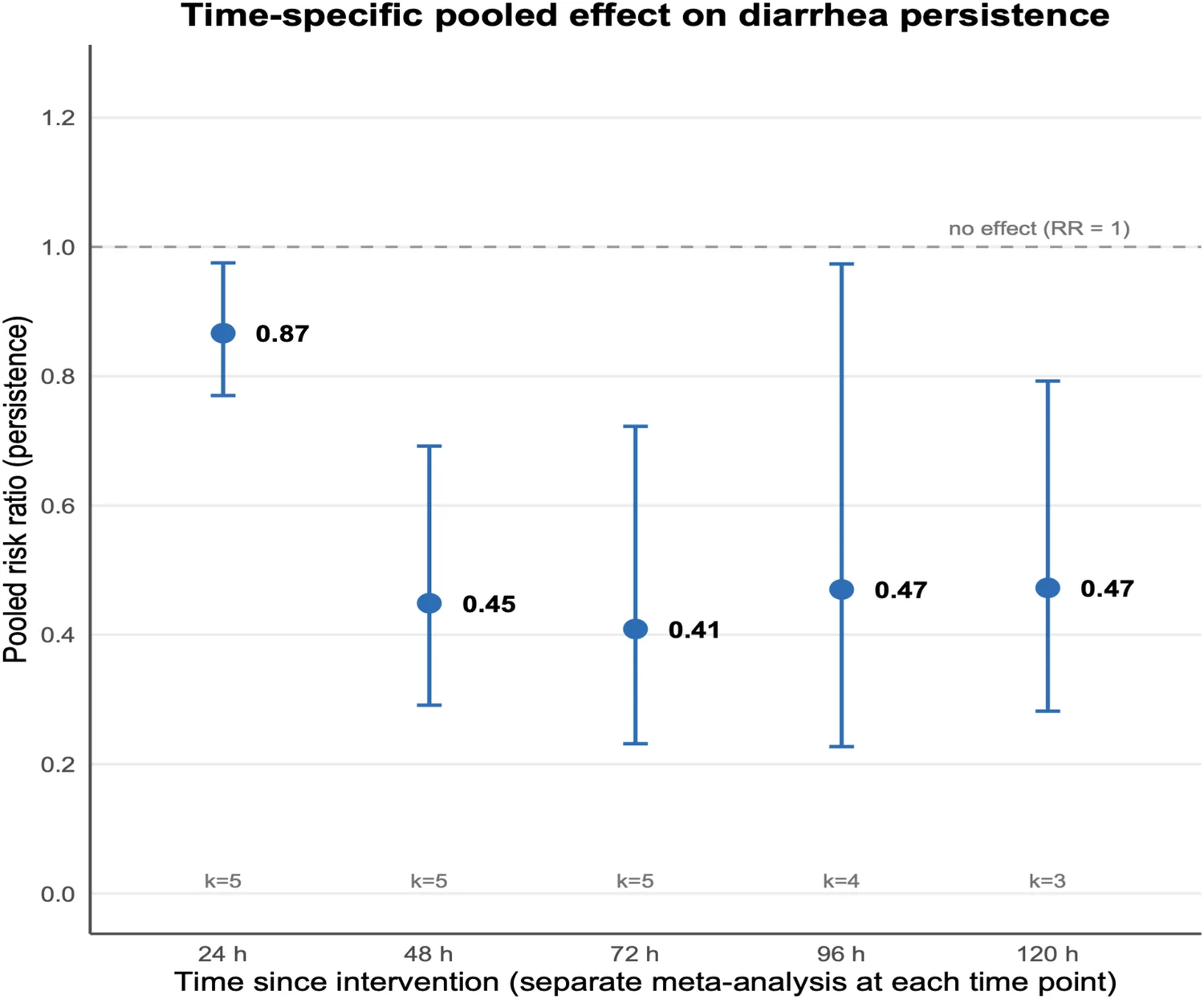
Time-specific pooled effect of L. reuteri DSM 17938 on diarrhea persistence. Each point represents a separate random-effects meta-analysis of the proportion of children with persisting diarrhea at that time point, with its 95% confidence interval; the estimates are not derived from a single longitudinal model, and the number of contributing trials differs across time points (k = 5 at 24–72 h; k = 4 at 96 h; k = 3 at 120 h, as one trial reported outcomes only up to 72 h and another contributed no events at 120 h). No connecting line is shown, as these are independent estimates rather than a modeled trajectory over time. RR < 1 favors L. reuteri DSM 17938.

#### Duration of hospitalization

Only two trials provided data suitable for pooling (a third reported no significant difference without extractable statistics). The pooled estimate was not statistically significant (MD −0.70 days, 95% CI −1.50 to 0.09; I^2^ = 87%).

#### Number of watery stools per day

Based on two trials, *L. reuteri* DSM 17938 was associated with fewer watery stools on day 2 (MD −1.49, 95% CI −2.23 to −0.75) and day 3 (MD −2.33, 95% CI −2.69 to −1.97).

#### Adverse events

No serious or intervention-related adverse events were reported in any of the six trials. In four trials, no adverse events considered related to the intervention were recorded (20–22, 24). In the trial by Szymański et al. (23), three events occurred in the *L. reuteri* DSM 17938 group (otitis media, hepatitis, and pneumonia) and one in the control group (otitis media), all judged unrelated to the study product. In the trial by Rerksuppaphol et al. (25), transient vomiting was reported in two children in the *L. reuteri* DSM 17938 group and one child in the control group. Overall, where adverse events were recorded, their frequency was comparable between the L. reuteri DSM 17938 and control groups, and no safety concerns were identified.

### Certainty of evidence

The GRADE evidence profile is summarized in Table 2. The certainty of evidence for the primary outcome (duration of diarrhea) was rated very low, with downgrades for risk of bias (a majority of trials at some concerns, with much of the pooled weight carrying blinding or reporting concerns), inconsistency (I2 = 79%), and imprecision (the confidence interval crossed a minimal clinical important difference of approximately one day). For the diarrhea-persistence outcomes at 48 and 72 h, the certainty was rated low: these outcomes were downgraded for risk of bias and inconsistency, but not for imprecision, as their confidence intervals lay entirely within the region of benefit (risk ratio well below 1). The certainty for duration of hospitalization was rated very low, downgraded for risk of bias, serious inconsistency (I2 = 87%), and imprecision arising from a non-significant estimate based on only two trials. For the assessment of imprecision in the primary and hospitalization outcomes, we used a minimal clinically important difference (MCID) of approximately one day in the duration of diarrhea. This threshold reflects the effect size around which the clinical relevance of probiotics for acute gastroenteritis has conventionally been judged: reductions of about one day have been the benchmark reported in prior syntheses and guideline discussions [9], and previous large, randomized trial in this field demonstrated that statistically detectable effects smaller than this may lack clear clinical importance (39). A confidence interval spanning from a trivial effect to approximately one day was therefore considered to cross a clinically important threshold, warranting a downgrade for imprecision. No formal certainty rating was assigned to adverse events, as no serious or intervention-related events occurred in any trial and the data were not suitable for pooling. Publication bias was not formally assessed for any outcome because fewer than 10 studies were available.

**Table 2:** GRADE summary of findings.

Outcome	No. studies	Effect (95% CI)	Heterogeneity	Certainty (GRADE)	Comment
Duration of diarrhea *(primary)*	6 RCTs	MD −0.68 days [−1.09, −0.27]	I² = 79%	⊕◯◯◯ **Very low**^a^^,^^b^^,^^c^	≈16 h shorter; clinical importance uncertain relative to a ∼1-day MCID
Diarrhea persistence at 24 h	5 RCTs	RR 0.87 [0.80, 0.95]	I² = 34%	⊕⊕⊕◯ **Moderate**^a^	Small early reduction; consistent across trials (low heterogeneity), CI within benefit
Diarrhea persistence at 48 h	5 RCTs	RR 0.45 [0.29, 0.69]	I² = 74%	⊕⊕◯◯ **Low**^a^^,^^c^	Maximal effect; CI entirely within benefit (no imprecision downgrade)^f^
Diarrhea persistence at 72 h	5 RCTs	RR 0.41 [0.23, 0.72]	I² = 75%	⊕⊕◯◯ **Low**^a^^,^^c^	CI entirely within benefit^g^
Diarrhea persistence at 96 h	4 RCTs	RR 0.47 [0.23, 0.95]	I² = 71%	⊕◯◯◯ **Very low**^a^^,^^c^^,^^d^	Wide CI, upper bound approaches 1.0
Diarrhea persistence at 120 h	3 RCTs	RR 0.47 [0.30, 0.73]	I² = 18%	⊕⊕◯◯ **Low**^a^^,^^e^	Attenuated but persistent benefit; sparse data
Duration of hospitalization	2 RCTs	MD −0.70 days [−1.50, 0.09]	I² = 87%	⊕◯◯◯ **Very low**^a^^,^^c^^,^^d^	Not statistically significant; sparse
Watery stools/day (day 2; day 3)	2 RCTs	Day 2: MD −1.49 [−2.23, −0.75]; Day 3: MD −2.33 [−2.69, −1.97]	NR	⊕⊕◯◯ **Low**^a^^,^^e^	Based on two trials only
Adverse events	6 RCTs	Not pooled	—	—	No serious or intervention-related events; frequency similar between groups

CI, confidence interval; MCID, minimal clinically important difference; MD, mean difference; NR, not reported; RCT, randomized controlled trial; RR, risk ratio. A risk ratio < 1 favors L. reuteri DSM 17938. The GRADE certainty of evidence is expressed as high (⊕⊕⊕⊕), moderate (⊕⊕⊕◯), low (⊕⊕◯◯) or very low (⊕◯◯◯).

a Risk of bias (−1): the majority of trials were rated “some concerns” on RoB 2 (two single-blind trials; two without prospective registration), and much of the pooled weight carries blinding or reporting concerns.

b Imprecision (−1, primary outcome): the 95% CI spans from a clinically trivial effect to approximately one day and borders the null under the Hartung–Knapp adjustment, crossing a minimal clinically important difference of ∼1 day (see Methods/Discussion for basis).

c Inconsistency (−1): substantial statistical heterogeneity, only partly explained by blinding and clinical setting.

d Imprecision (−1): wide 95% CI (approaching or crossing the null) based on few trials.

e Imprecision (−1): estimate derived from only two to four small trials (sparse data).

f Not downgraded for imprecision: the 95% CI lies entirely within the region of benefit (does not cross the null).

Publication bias was not assessed because fewer than 10 trials were available; certainty was not downgraded on this domain for any outcome.

## Discussion

This updated systematic review and meta-analysis of six randomized controlled trials found that, compared with placebo or no treatment, *L. reuteri* DSM 17938 significantly reduced the duration of diarrhea in children with acute gastroenteritis by a mean difference of 0.68 days (approximately 16 h). The effect on diarrhea persistence was greatest at 48 to 72 h after the start of treatment. No serious or intervention-related adverse events were reported in any trial. However, the magnitude of the effect on the primary outcome was small, heterogeneity was high, and the overall certainty of evidence was very low.

Our findings extend and refine the previous four-trial meta-analysis, which reported a reduction in diarrhea duration of 0.87 days (95% CI −1.43 to −0.31) (19). By adding two double-blind trials conducted during and after the COVID-19 pandemic (24, 25), the pooled estimate moved toward the null (−0.68 days), while remaining statistically significant. Importantly, restricting our analysis to the original four trials reproduced the earlier estimate exactly, confirming that the shift reflects the contribution of the new trials rather than differences in method. Our updated estimate is also concordant with the meta-analysis embedded in the 2025 Ibero–Latin American clinical practice guideline, which reported a pooled reduction of 0.87 days overall and −0.68 days in the subgroup of adequately blinded trials — a value essentially identical to our overall result (18). The convergence of three independent analyses on a small, sub–one-day effect strengthens confidence in the point estimate, even as its clinical relevance remains uncertain.

A central observation of this review is the concordance between blinding, risk of bias, and effect size. The two single-blind trials (20, 21) — rated at some concerns for the subjective, caregiver-ascertained outcome — produced the largest effects, whereas the four double-blind trials yielded approximately half the magnitude (−0.42 vs. −0.99 days) with markedly lower heterogeneity (I^2^ = 37% vs. 86%). The formal test for subgroup differences was not statistically significant (*p* = 0.19), an expected limitation given only six trials. Nonetheless, this pattern is consistent with performance and detection bias inflating subjective outcomes in trials without participant blinding, in line with the blinding-restricted sensitivity analyses reported in the 2019 meta-analysis and the 2025 regional guideline (18, 19). Because this subgroup comparison was not statistically significant and was based on only six trials, it should be regarded as exploratory and hypothesis-generating. Nonetheless, the pattern is consistent with the well-documented tendency of unblinded trials to inflate subjective outcomes, and the double-blind estimate (−0.42 days) may provide a more conservative, and possibly less biased, reflection of the effect. This interpretation should be viewed as suggestive rather than definitive.

The analysis of diarrhea persistence across successive time points added a coherent picture that was not available from the duration outcome alone. The benefit was small at 24 h, reached its maximum at 48 to 72 h (with roughly a halving of the risk of persisting diarrhea), and then attenuated as most children in both groups recovered. This temporal pattern is consistent with the early-recovery advantage reported in individual trials and in the regional guideline analysis and suggests that any clinical benefit of *L. reuteri* DSM 17938 is concentrated in the first two to three days of illness.

A distinctive feature of this update is the inclusion of two trials conducted during and after the COVID-19 pandemic, a period marked by substantial changes in the epidemiology of pediatric acute gastroenteritis, including altered circulation of rotavirus and norovirus and shifts in causative pathogens (3–5). In an exploratory analysis, the two post-pandemic trials showed a smaller and more homogeneous effect (MD approximately −0.38 days; I^2^ = 0%) than the pre-pandemic trials (24, 25). This observation is compatible with the hypothesis that a changing etiological landscape may modify probiotic efficacy. However, it must be interpreted with considerable caution: the post-pandemic period coincides almost entirely with double-blind design, non-European setting, and lower dose, so the apparent “period effect” cannot be disentangled from these factors, and it rests on only two trials. Moreover, the two post-pandemic trials were themselves conducted in different epidemiological phases — one during the period of suppressed viral circulation and one during the subsequent rebound — further limiting inference. This finding should therefore be regarded as hypothesis-generating, underscoring the need for future trials with systematic an etiological characterization.

Our findings should also be viewed alongside evidence on *L. reuteri* DSM 17938 for outcomes beyond the acute diarrheal episode. In a multicenter, randomized, factorial trial in children hospitalized in Botswana with acute gastroenteritis, a 60-day course of *L. reuteri* DSM 17938 (1 × 10⁸ CFU/day) did not improve linear growth (adjusted height-for-age z-score at 60 days: mean difference −0.07 SD, 95% CI −0.22 to 0.08) or other important outcomes, including recurrent diarrhea, mortality, or length of hospital stay (36). That trial addressed a different primary outcome (growth rather than symptom duration), used a markedly longer treatment course, and enrolled children in a high-burden, resource-limited setting with a high prevalence of enteropathogens. Despite these differences, its predominantly null point estimates are broadly consistent with the modest and uncertain benefit observed in our analysis. Together, these data suggest that any effect of *L. reuteri* DSM 17938 in childhood acute gastroenteritis is likely to be small and largely confined to the acute phase of illness, without clear medium-term or growth-related benefit. Evidence from the antibiotic-associated diarrhea (AAD) setting provides complementary support. In the PEARL study — a large, multicenter, double-blind, placebo-controlled trial in children treated in outpatient settings receiving amoxicillin-clavulanate. — *L. reuteri* DSM 17938 (2 × 10⁸ CFU/day) significantly reduced the incidence of AAD at 14, 21 and 56 days (35). Notably, among children who did develop diarrhea, the mean duration was shorter in the *L. reuteri* DSM 17938 group than in the placebo group (2.8 ± 0.99 vs. 3.2 ± 0.93 days; *p* < 0.05) — a magnitude of effect (approximately 0.4 days) closely consistent with the pooled reduction observed in the present meta-analysis of acute infectious gastroenteritis. Although AAD and acute infectious gastroenteritis are distinct clinical entities, the convergence of these independent datasets on a similar, sub–one-day shortening of diarrhea duration lends coherence to the overall picture: where *L. reuteri* DSM 17938 shortens diarrhea, the effect is real but modest in size.

The mechanisms by which *L. reuteri* DSM 17938 may shorten acute diarrhea are not fully established but are thought to include the production of reuterin, a broad-spectrum antimicrobial compound that inhibits enteropathogen growth; competition with pathogens for adhesion sites and nutrients; strengthening of the intestinal barrier through enhanced tight-junction integrity and mucus production; and immunomodulation, including stimulation of secretory IgA (11, 16, 40). By these routes, *L. reuteri* DSM 17938 may accelerate the resolution of the gut dysbiosis that accompanies acute infectious diarrhea. In children with acute infectious diarrhea, the transition from a diseased to a healthy state is mirrored by a recovery of gut-microbiota diversity and configuration over time providing a biological rationale for microbiota-targeted adjuncts (41). Nevertheless, the extent to which probiotic-induced changes in microbiota translate into clinically meaningful shortening of diarrhea remains incompletely understood, consistent with the modest effect size observed in the present analysis.

The clinical effect of *L. reuteri* DSM 17938 may also carry economic implications. In a cost analysis based on multicenter trials in Türkiye, the addition of *L. reuteri* DSM 17938 to standard care reduced the direct costs of acute gastroenteritis In children who are hospitalized (203 ± 65 vs. 254 ± 68 US) and in those attending emergency care (53.4 ± 17.8vs79.6 ± 20US), primarily through shorter hospital and emergency-care stays; when extrapolated to the annual burden of rotavirus gastroenteritis, hospitalization- and emergency-related costs were reduced by approximately 28% (42). In the outpatient setting, by contrast, the direct cost was slightly higher in the probiotic group, reflecting the added cost of the product without an offsetting reduction in hospital stay. These preliminary economic data suggest that any benefit of *L. reuteri* DSM 17938 may be most relevant in inpatient and emergency settings, although a full assessment incorporating indirect and societal costs is needed. Such considerations should be weighed against the modest and low-certainty effect on diarrhea duration demonstrated in the present analysis.

### Strengths

This review used a validated extraction and analysis pipeline (confirmed by exact reproduction of the previous pooled estimate), applied a formal Risk of Bias 2 assessment and GRADE rating, examined a full time-course of diarrhea persistence, and undertook prespecified subgroup and sensitivity analyses. The focus on a single, well-characterized strain avoids the heterogeneity inherent in pooling different probiotic products and aligns with the strain-specific principle endorsed by current guidelines.

### Limitations

The number of trials was small, and heterogeneity was high and only partly explained by blinding and setting. Two trials reported the primary outcome as medians and required conversion to means and standard deviations, although sensitivity analyses showed the pooled estimate to be robust to this. The most methodologically conservative trial did not report the diarrhea-persistence outcome in a usable form and was excluded from those analyses, which may have shifted the pooled risk ratios slightly toward benefit. Definitions of the resolution of diarrhea differed across trials, and analyses were based on available-case data rather than a uniform intention-to-treat approach. Although the DerSimonian–Laird estimator can underestimate uncertainty with few trials, the concordance with the more conservative Hartung–Knapp analysis supports the robustness of our conclusions. Finally, because fewer than 10 trials were available, publication bias could not be assessed formally.

Funding sources and conflict-of-interest disclosures of the included trials are summarized in Table 1. Notably, none of the six trials reported direct industry funding: two were investigator-initiated (20, 21), one was supported by a university research grant (25), one used study product supplied by the manufacturer while explicitly stating that the manufacturer had no role in the study (23), and the remaining trials reported no funding (22, 24). Although some investigators—including authors of the present review—have disclosed professional relationships with probiotic manufacturers, the limited degree of direct industry involvement in the included trials reduces, though does not eliminate, the concern that the pooled estimate might be inflated by funding-related bias. This consideration is nonetheless consistent with our cautious interpretation and with the observation that the largest effects arose in trials at greater risk of bias.

Taken together, the evidence indicates that *L. reuteri* DSM 17938 produces a small, probably genuine reduction in the duration of childhood acute gastroenteritis and a moderate reduction in diarrhea persistence at 48 to 72 h, with a favorable safety profile, but that the effect on the primary outcome is of uncertain clinical importance and rests on very-low-certainty evidence. These findings are consistent with the current conditional (weak) recommendations for *L. reuteri* DSM 17938 in pediatric acute gastroenteritis, while underscoring that the supporting evidence is of low to very low certainty. Any decision about its clinical use rests with guideline panels applying a full evidence-to-decision framework; our results provide the updated evidence base to inform, rather than to issue, such recommendations. They also help explain the divergent certainty ratings attached to this strain across guidelines, which reflect the limited and heterogeneous underlying data. Future research should prioritize adequately powered, double-blind trials with systematic pathogen characterization, standardized definitions of diarrhea resolution, and intention-to-treat analysis, ideally conducted in the current post-pandemic epidemiological context, to clarify whether — and in which subgroups — *L. reuteri* DSM 17938 offers clinically meaningful benefit. Collectively, these findings underscore the need for a new generation of adequately powered, rigorously designed trials of *L. reuteri* DSM 17938 in pediatric acute gastroenteritis, planned and reported in accordance with contemporary methodological standards for probiotic research. The recently proposed Probiotic Human Study Design and Reporting (PRODARE) recommendations offer a practical framework for this purpose (43). They emphasize precise identification and characterization of the probiotic strain, transparent reporting of the administered dose and viable count throughout the study, prospective registration, prespecified and standardized clinical outcomes, and analysis according to the intention-to-treat principle. Applying these standards would directly address the principal limitations identified in the present synthesis — most notably the reliance on subjective, caregiver-ascertained endpoints in inadequately blinded trials, the heterogeneous definitions of diarrhea resolution, and the use of available-case rather than intention-to-treat data. Beyond reporting, future trials should be double-blind and placebo-controlled, adequately powered against a prespecified minimal clinically important difference, and should use uniform, objective definitions of diarrhea onset and resolution. They should also incorporate systematic etiological characterization to account for the shifting post-pandemic epidemiology of enteric pathogens. Only trials of this quality will be able to establish whether the small, sub–one-day benefit suggested by the current evidence represents a reproducible and clinically meaningful effect, and to identify the subgroups — by pathogen, setting, or dose — in which it is most likely to matter.

## Conclusion

In this updated meta-analysis, *L. reuteri* DSM 17938 significantly but modestly reduced the duration of diarrhea and the persistence of diarrhea at 48 to 72 h in children with acute gastroenteritis, with a good safety profile. The magnitude of the effect on the primary outcome was small and of uncertain clinical importance, heterogeneity was high, and the certainty of evidence was very low, with the benefit concentrated in single-blind trials. These findings are consistent with the conditional role currently assigned to *L. reuteri* DSM 17938 in the treatment of pediatric acute gastroenteritis and highlight the need for higher-quality, etiology-informed trials.

## Data Availability

The data analyzed in this study is subject to the following licenses/restrictions: This study did not generate original datasets. The analyses were based on data extracted from previously published studies. No independent dataset was created or deposited as part of this work. Requests to access these datasets should be directed to dimasrosa@gmail.com.
