# Supplementary material for: Efficacy and safety of Limosilactobacillus reuteri DSM 17938 for the treatment of acute gastroenteritis in children: a systematic review and meta-analysis of randomized controlled trials
Source: Front Pediatr. 2026 Oct 16;14:1941872. doi: 10.3389/fped.2026.1941872 (PMC13719600; doi:10.3389/fped.2026.1941872)
Supplement: Supplementary file 1 [file Supplementaryfile1.docx]

**Supplementary Figure 1.** Risk of bias assessment of the included randomized controlled trials using the Cochrane Risk of Bias 2 (RoB 2) tool.

Percentages represent the proportion of the six included randomized controlled trials judged as having low risk of bias or some concerns for each RoB 2 domain and overall assessment. The plot was generated using **robvis**.

**Supplementary Table 1. Full electronic search strategy by database.**

Search date: [01/04/2026]. No language or date restrictions were applied unless stated. Concept blocks: (A) intervention, (B) condition, (C) population.

- **MEDLINE (PubMed); records retrieved: 199**

A ("*Limosilactobacillus reuteri*"[tiab] OR "*Lactobacillus reuteri*"[tiab] OR "*L reuteri*"[tiab] OR "DSM 17938"[tiab] OR "DSM17938"[tiab])

AND

B ("diarrhea"[MeSH Terms] OR diarrhoea[tiab] OR diarrhea[tiab] OR "gastroenteritis"[MeSH Terms] OR gastroenteritis[tiab])

AND

C ("child"[MeSH Terms] OR "infant"[MeSH Terms] OR child*[tiab] OR infant*[tiab] OR pediatric*[tiab] OR paediatric*[tiab])

- **Embase; records retrieved: 212**

A (*'Limosilactobacillus reuteri’: ti*,ab,kw OR *'Lactobacillus reuteri'*:ti,ab,kw OR 'DSM 17938':ti,ab,kw OR 'DSM17938':ti,ab,kw)

AND

B ('diarrhea'/exp OR diarrhea:ti,ab,kw OR diarrhoea:ti,ab,kw OR 'gastroenteritis'/exp OR gastroenteritis:ti,ab,kw)

AND

C ('child'/exp OR 'infant'/exp OR child*:ti,ab,kw OR infant*:ti,ab,kw OR pediatric*:ti,ab,kw OR paediatric*:ti,ab,kw)

- **Scopus; records retrieved: 296**

A TITLE-ABS-KEY("*Limosilactobacillus reuteri*" OR "*Lactobacillus reuteri*" OR "DSM 17938" OR "DSM17938")

B AND TITLE-ABS-KEY (diarrhea OR diarrhoea OR gastroenteritis)

C AND TITLE-ABS-KEY(child* OR infant* OR pediatric* OR paediatric*)

- **Web of Science (Science Citation Index Expanded)**: records retrieved: 123

TS=("*Limosilactobacillus reuteri*" OR "*Lactobacillus reuteri*" OR "DSM 17938" OR "DSM17938")

AND TS=(diarrhea OR diarrhoea OR gastroenteritis)

AND TS=(child* OR infant* OR pediatric* OR paediatric*)

- **ClinicalTrials.gov**; records retrieved: 23

Condition/disease: diarrhea OR gastroenteritis

Other terms: *Lactobacillus reuteri* DSM 17938; *Limosilactobacillus reuteri DSM 17938*

- Google Scholar; first 200 records screened by relevance

"*Lactobacillus reuteri*" "DSM 17938" “*Limosilactobacillus reuteri*” (diarrhea OR gastroenteritis) children
